# Systems-Based Identification of Temporal Processing Pathways during Bone Cell Mechanotransduction

**DOI:** 10.1371/journal.pone.0074205

**Published:** 2013-09-11

**Authors:** Leah E. Worton, Brandon J. Ausk, Leah M. Downey, Steven D. Bain, Edith M. Gardiner, Sundar Srinivasan, Ted S. Gross, Ronald Y. Kwon

**Affiliations:** Department of Orthopaedics and Sports Medicine, University of Washington, Seattle, Washington, United States of America; University of Notre Dame, United States of America

## Abstract

Bone has long been established to be a highly mechanosensitive tissue. When subjected to mechanical loading, bone exhibits profoundly different anabolic responses depending on the temporal pattern in which the stimulus is applied. This phenomenon has been termed temporal processing, and involves complex signal amplification mechanisms that are largely unidentified. In this study, our goal was to characterize transcriptomic perturbations arising from the insertion of intermittent rest periods (a temporal variation with profound effects on bone anabolism) in osteoblastic cells subjected to fluid flow, and assess the utility of these perturbations to identify signaling pathways that are differentially activated by this temporal variation. At the level of the genome, we found that the common and differential alterations in gene expression arising from the two flow conditions were distributionally distinct, with the differential alterations characterized by many small changes in a large number of genes. Using bioinformatics analysis, we identified distinct up- and down-regulation transcriptomic signatures associated with the insertion of rest intervals, and found that the up-regulation signature was significantly associated with MAPK signaling. Confirming the involvement of the MAPK pathway, we found that the insertion of rest intervals significantly elevated flow-induced p-ERK1/2 levels by enabling a second spike in activity that was not observed in response to continuous flow. Collectively, these studies are the first to characterize distinct transcriptomic perturbations in bone cells subjected to continuous and intermittent stimulation, and directly demonstrate the utility of systems-based transcriptomic analysis to identify novel acute signaling pathways underlying temporal processing in bone cells.

## Introduction

Temporal processing is the process by which cells perceive temporal variations of an applied stimulus. While this process is most commonly associated with neural functions such as auditory processing [1], a growing body of evidence suggests that diverse cell types exhibit temporal processing when exposed to a broad range of stimuli. For example, the literature is replete with cases in which the insertion of rest periods during administration of a particular stimulus results in enhanced or even opposite (i.e., sensitization versus tolerance) effects, despite the same magnitude of stimulus being applied [2], [3], [4], [5], [6]. Interestingly, this phenomenon has been observed in response to diverse signals including pharmacological [3], electrophysiological [4], biochemical [5], and mechanical stimuli [6], suggesting the existence of conserved signaling mechanisms that enable temporal processing at the cellular level.

Bone has long been established to be a highly mechanosensitive tissue, capable of undergoing rapid and robust bone formation in response to microscopic deformations [7]. Given that mechanical loading is one of the primary determinants of bone strength, the mechanotransduction pathway is widely recognized as a promising target for new bone therapeutic strategies [8], [9], [10]. During mechanotransduction, bone exhibits temporal processing in a manner that profoundly affects its anabolic response to mechanical loading [6], [11], [12], [13], [14]. For example, it has been previously shown that selectively removing mechanical signals via insertion of 10 s rest intervals has the potential to transform a low magnitude, non-osteogenic cyclic loading regimen into a potent anabolic signal in mice, despite a ten-fold decrease in the number of load cycles [6]. In neural research, this form of temporal processing is referred to as temporal unmasking [15], i.e., heightened perception of a stimulus when presented in a particular temporal pattern. Though significant efforts have been made to elucidate the molecular underpinnings of temporal processing in bone, this phenomenon remains poorly understood. For example, only one pathway (enhanced intracellular Ca2+ mobilization [16], [17], [18], [19]) has been implicated in this process in the last twenty years.

Investigation of the mechanistic basis of temporal processing presents several unique and fundamental challenges. For example, this phenomenon involves the coordinated actions of multiple signaling pathways, with the predominance of a particular pathway dictated by the temporal pattern of stimulation [12]. In accordance with this, in bone, both short- (on the order of seconds) and long- (on the order of tens of minutes to hours) duration rest intervals have been found to enhance loading-induced adaptation, with distinct molecular mechanisms suggested to underlie their anabolic effects [12]. The potential involvement of multiple pathways makes the systematic interrogation of this process highly challenging, as it requires probing a spectrum of signaling pathways within a single experimental framework. A second challenge is the fact that temporal processing relies on the amplification of subtle variations in signaling network dynamics. Such amplification can occur, for example, through the cumulative effect of many small perturbations in signaling dynamics as they are propagated through an interconnected signaling network. In this case, at the level of gene expression, acute cell responses arising from temporal variations in stimulation may be very subtle, with the causal pathways driving these small transcriptional changes unapparent when observed in a single gene in isolation.

In this study, our goal was to characterize transcriptomic perturbations arising from the insertion of intermittent rest periods in bone cells subjected to fluid flow, and assess the utility of these perturbations to identify signaling pathways that are differentially activated by this temporal variation. At the level of the genome, we found that the common and differential alterations in gene expression arising from the two flow conditions were distributionally distinct, with the differential alterations characterized by many small changes in a large number of genes. Using bioinformatics analysis, we identified distinct up- and down-regulated gene expression signatures emerging from the insertion of rest intervals, and found that the up-regulation signature was significantly associated with MAPK signaling. Finally, we validated this observation by demonstrating that rest intervals significantly elevated flow-induced activity of the MAPK ERK1/2 by enabling a second spike in phosphorylation that was not observed in response to continuous flow. Together, these studies are the first to characterize transcriptomic perturbations in bone cells subjected to continuous and intermittent stimulation, and directly demonstrate the utility of systems-based transcriptomic analysis to identify novel acute signaling pathways underlying temporal signal processing in bone cells.

## Materials and Methods

### Cell Culture and Fluid Flow

**Figure 1:**
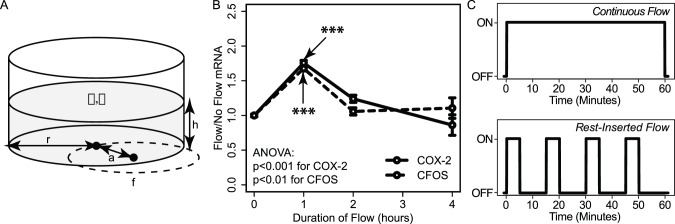
*In vitro* model of temporal processing during bone cell mechanotransduction. (A) Bone cells were subjected to fluid shear stress by subjecting them to orbital shaking. The schematic depicts quantities in Eq. 1 as well as others expected to influence shear stress. (B) Time course of COX-2 and CFOS gene expression in bone cells subjected to continuous orbital shaking of different durations. Increased expression is observed after one hour of flow for both genes, indicating that the shear stress generated via orbital flow was sufficient to stimulate acute gene expression. ***p<0.001 for flow versus no flow. (C) Schematic demonstrating flow profiles for continuous and rest-inserted flow. Rest-inserted flow consisted of 5 min of flow followed by 10 min of rest, repeated four times.

MC3T3-E1 osteoblastic cells (clone 14, passage 9–12) were cultured on tissue culture plates in growth media (α-MEM with 10% FBS) at 37°C and 5% CO_2_ [20]. Seventy-two hours prior to experimentation, cells were seeded into 6-well plates (Corning) at a density of 2.5×10^4^ cells per well in 2 ml of growth media. For flow experiments, plates were carefully transferred to an orbital shaker (VWR, DS-500) placed in an incubator at 37°C and 5% CO_2_ one hour prior to experimentation. To generate flow, plates were subjected to orbital shaking (2.2 Hz), which resulted in a rotating wave on the fluid surface and dynamic shear stress on the bottom of the well [21]. While the use of parallel plate chambers has several advantages over the use of orbital shaking for the generation of fluid flow (e.g., the ability to apply a well-defined shear stress and to decouple flow velocity from flow frequency [22]), we used orbital shaking to alleviate the potential for inadvertent activation of acute signaling pathways that can occur during loading of cells into the flow chambers [23]. Shear stress was estimated using an approximate relation for the maximal wall shear stress [24]:
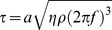
(1)where *τ* is the shear stress, *a* is the orbital radius, *η* is the dynamic viscosity, *ρ* is the fluid density, and *f* is the orbital frequency. Using appropriate values for our experimental setup (*f* = 2.2 Hz, *a* = 0.95 cm, η = 0.01P, and ρ = 1.0 g/ml, see Fig. 1A), we computed *τ* to be ∼0.5Pa. It is important to note that Eq. 1 is only a first-order approximation of the shear stress. This is evident by the fact that shear stress is expected to depend on fluid height above the cell surface as well as the radius of the well [25], which are not accounted for in Eq. 1. Given that a peak shear stress of ∼0.1Pa has been previously found experimentally using identical shaking parameters to those used in this study [26], we estimate that peak shear stresses in our system were in the range of 0.1∼0.5Pa.

### Real-Time RT-PCR

For real-time RT-PCR, total RNA was extracted using the RNeasy Mini Kit (Qiagen) according to manufacturer’s protocol. cDNA was synthesized using Super Script III reverse transcriptase (Invitrogen), and real-time PCR was performed using SYBR green and the Applied Biosystems 7900 HT sequence detection system (see Table S1 for primer sequences for each gene). Gene expression levels were quantified using the 2^∧^(−ΔΔC_T_) method [27], with either cyclophilin or beta-actin as the housekeeping gene depending on the experiment.

### Western Blot

To assess ERK1/2 activity, total cellular protein was isolated in RIPA lysis buffer (50 mM Tris-HCl pH 8, 150 mM NaCl, 1% NP-40, 0.5% sodium deoxycholate, 0.1% SDS), separated by electrophoresis in 4–12% NuPAGE Bis-Tris gels (Invitrogen, Life Technologies) and transferred onto PVDF membranes (GE Healthcare). Membranes were probed with rabbit p-ERK and ERK antibodies (Cell Signaling Technology, 1∶1000). Bound primary antibodies were detected by chemiluminescent detection of HRP-conjugated donkey anti-rabbit IgG antibody (GE Healthcare, 1∶5000).

### Microarray Analysis

For microarray analysis, RNA was isolated using procedures identical to those described for RT-PCR. RNA was isolated from cells exposed to one of three different flow conditions (no flow, rest-inserted flow, and continuous flow) and one of two different time points (immediately following cessation of flow and one hour post-flow). This resulted in six experimental conditions, henceforth referred to as NF_0_, NF_1_, RF_0_, RF_1_, CF_0_, and CF_1_. For each of the six experimental conditions, RNA samples were pooled from three independent experiments (18 individual samples total). Frozen samples were submitted to the Genetics Core Facility at Benaroya Research Institute and analyzed using MouseWG-6 v2.0 Beadarray Chips (Illumina). Briefly, RNA integrity was measured using a Bioanalyzer 2100 (Agilent), and concentration was assessed using a NanoQuant (Tecan). cRNA was prepared by amplification and labeling using the Illumina TotalPrep RNA Amplification Kit (Life Technologies) and hybridized to Beadarray Chips. Beadchips were scanned on a HiScanSQ (Illumina). Background subtracted data was generated using GenomeStudio Software (Illumina). Data were pre-processed by performing quantile normalization, flooring (raw intensity values less than 10 were set to 10), log2 transformation, and filtering (for each probe, at least one sample was required to have a p-value of less than 0.01). Of the 45,281 genes/probes within the array, 15,686 (34.6%) were found to meet pre-processing filtering criteria. For simplicity, we further filtered out an additional 3716 genes consisting of unnamed RIKEN cDNA genes and probes in which gene symbols were not available, resulting in a final total of 11,970 different genes/probes for analysis. Background subtracted and pre-processed data have been submitted to the Gene Expression Omnibus under the series accession number GSE48177.

### Data Transformation

Using gene expression profiles obtained from the six experimental conditions (NF_0_, NF_1_, RF_0_, RF_1_, CF_0_, and CF_1_), we computed the log-transformed flow/no flow ratios for cells subjected to continuous and rest-inserted flow for each time point as.
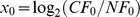


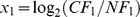


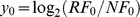


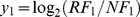
(2)


The time-averaged values of the continuous and rest-inserted flow/no flow ratios were computed as.
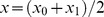


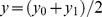
(3)


### Data Projection

For analysis, microarray data were projected into a two-dimensional Euclidean gene expression space denoted as (*x*,*y*) space. Within this space, each gene was projected as a point using its *x* and *y* values as position coordinates. For each point, the *x* coordinate measured its time-averaged fold change in expression following exposure to continuous flow, while the *y* coordinate measured the same in response to rest-inserted flow. In this case, any gene that exhibited identical expression under the two flow conditions laid along the diagonal *y* = *x*. Conversely, for any gene not on the diagonal, its distance from the line *y* = *x* measured its differential expression arising from the insertion of rest intervals. To measure this distance, we also defined a second coordinate system by rotating the original coordinate system by 45° as.
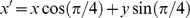


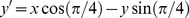
(4)


For each gene (*x*,*y*), its *x*’ coordinate scaled with distance along the line *y* = x and measured its common alterations in expression under the two flow conditions. In contrast, its *y*’ coordinate scaled with distance from the line *y* = *x* and measured its differential expression under the two flow conditions.

### Data Analysis

All data analysis was performed in the open source statistical environment R (http://www.R-project.org/). Hierarchical clustering of real time RT-PCR data was performed using Ward’s method. Analysis of microarray data was performed using the lumi package within the Bioconductor framework [28]. Computation of kernel density estimates and relative kernel density estimates was performed using the density function and the reldist package, respectively. GO (Gene Ontology) and KEGG (Kyoto Encyclopedia of Genes and Genomes) analyses were performed using GATHER (Gene Annotation Tool to Help Explain Relationships) [29] with mouse as the selected organism and network inference enabled (Bayes Factor>6 was considered significant). For SPEED (Signaling Pathway Enrichment using Experimental Data sets) analysis, we used the default settings (max absolute z-score percentile: 1%, min percent overlap across experiments: 20%, max expression level percentile: 50%) with all pathways enabled (FDR <0.05 was considered significant).

### Statistics

One-way ANOVA and Fisher’s PLSD post-hoc tests were performed for gene expression time course and p-ERK1/2 studies. Differences in distributions were assessed using a two-sample Kolmogorov-Smirnov test. Unless otherwise noted, p<0.05 was considered statistically significant.

## Results

### Rest-inserted and Continuous Fluid Flow Give Rise to Acute Variations in Gene Expression

We first implemented and validated an *in vitro* model of bone cell mechanotransduction that exhibited acute differences in gene expression in response to continuous and rest-inserted fluid flow. Cells were exposed to fluid flow by subjecting them to orbital shaking [23], [24]. We estimated that peak shear stresses in our system were in the range of 0.1∼0.5Pa, slightly lower than the 0.8–3.0Pa predicted to occur on the osteocyte cell process [30], [31] but significantly greater than those on the osteocyte cell body (due to the larger pericellular fluid space surrounding the cell body [32], [33]). To confirm the capacity for these relatively low levels of shear to stimulate bone cells, we assessed expression of the early response genes COX-2 [34] and CFOS [35] immediately following 1 h, 2 h, or 4 h of flow (Fig. 1B). We found that flow significantly increased expression of both genes (COX-2: p<0.001; CFOS: p = 0.003), with peak expression observed following one hour of flow for both genes (COX-2: p<0.001; CFOS: p<0.001). Exposure to longer duration flow was not found to be stimulatory. In particular, following one hour of flow, expression of both genes returned back to baseline levels, with no significant differences observed following two hours (COX-2: p = 0.053; CFOS: p = 0.61) or four hours (COX-2: p = 0.30; CFOS: p = 0.41) of flow.

Based on our results indicating that one hour of orbital flow was sufficient to stimulate acute gene expression, we next examined the effects of inserting rest intervals within the flow regimen. Motivated by previous studies indicating that rest intervals on the order of tens of minutes enhance bone adaptation to mechanical loading [12], we subjected cells to either one hour of continuous flow or rest-inserted flow consisting of four serial 15 min flow bouts, with each bout consisting of 5 min of flow followed by 10 min of rest (Fig. 1C).

**Figure 2:**
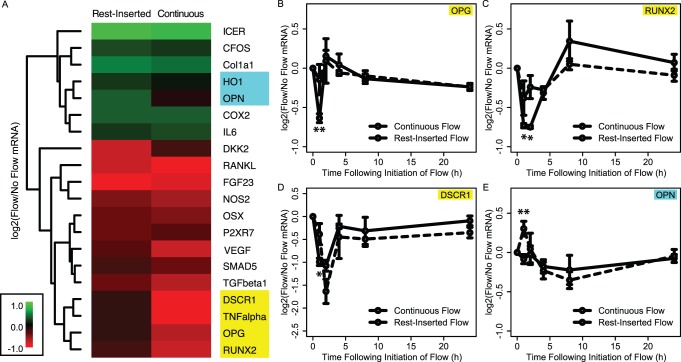
Continuous and rest-inserted fluid flow give rise to acute differences in gene expression that are highly transient. (A) Panel of 20 genes screened for differential expression immediately following cessation of continuous or rest-inserted flow. Though most of the genes assessed did not exhibit significant alterations by the insertion of rest intervals, we observed two sub-clusters (highlighted in blue and yellow) that exhibited detectable differences in expression following rest-inserted and continuous flow. Of these six genes, four were found to be significantly different under the two flow conditions: OPG, RUNX2, DSCR1, and OPN. (B–D) Time course of gene expression for these four genes following exposure to one hour of continuous or rest-inserted flow. Differences in expression observed immediately following continuous or rest-inserted flow were highly transient and tended to be focused within the 0–1 h period follow cessation of the flow. *p<0.05, **p<0.01, ***p<0.001 for rest-inserted flow vs. continuous flow.

We first performed a targeted RT-PCR screen to assess the potential for these two flow conditions to give rise to differential gene expression. In particular, using RNA harvested immediately following flow cessation, we assessed a panel of 20 genes selected based on their previous implication in the mechanotransduction (e.g., CFOS, COX-2, etc.) or bone anabolic (e.g., ALP, OSX, etc.) pathways. Consistent with previous studies, we found that most of the genes assessed did not exhibit significant alterations by the insertion of rest intervals [36]. However, two small gene clusters exhibited noticeable differences in expression following exposure to rest-inserted versus continuous flow (Fig. 2A). The first cluster of genes (DSCR1, TNF-alpha, OPG, and RUNX2) was down-regulated by continuous flow but not rest-inserted flow; the second cluster of genes (HO1 and OPN) was up-regulated by rest-inserted flow but not continuous flow. Of these six genes, four exhibited significant differences in expression between the two flow conditions (OPG: p = 0.002; RUNX2: p<0.05; DSCR1: p = 0.02; OPN: p = 0.003) and were subsequently subjected to time course analysis (0 h, 1 h, 3 h, 7 h, and 23 h post-flow). We found that differences in expression observed immediately following continuous or rest-inserted flow were not sustained over long durations. Rather, these changes tended to be highly transient and focused within the 0–1 h period follow cessation of the flow regimen (Fig. 2B–2E).

### Genome-wide Gene Expression Profiling Reveals Transcriptomic Variations Arising from the Insertion of Rest Intervals

We next sought to characterize variations in gene expression in cells subjected to rest-inserted versus continuous flow at the level of the genome. To do this, we performed genome-wide gene expression profiling using microarray analysis. In the time course studies described above, differential gene expression between continuous and rest-inserted flow predominated within the 0–1 h period following flow cessation. In this case, for microarray analysis we assessed gene expression at two time points: immediately following cessation of the one hour flow period, and one hour post-flow. Using the genome-wide gene expression profiles obtained from the microarray analysis, we computed log-transformed flow/no flow mRNA ratios for both time points (denoted as *x*_0_, *x*_1_, *y*_0_, and *y*_1_, see Eq. 2), as well time-averaged quantities (denoted as *x* and *y*, see Eq. 3). In our initial analysis, we found that the variances of the time-averaged quantities were approximately half those of the non-time averaged quantities, with σ^2^ = ∼4×10^−3^ for the former and σ^2^ = ∼8×10^−3^ for the latter. We interpreted the larger variance in the non-time averaged quantities to be due to genes that exhibited large fluctuations in expression between the two time points, which were subsequently smoothed by time-averaging. To increase the specificity of our analysis, we used the time-averaged profiles *x* and *y* for the rest of our studies, enabling us to focus our analysis on the identification of genes that exhibited sustained differences over both time points.

**Figure 3:**
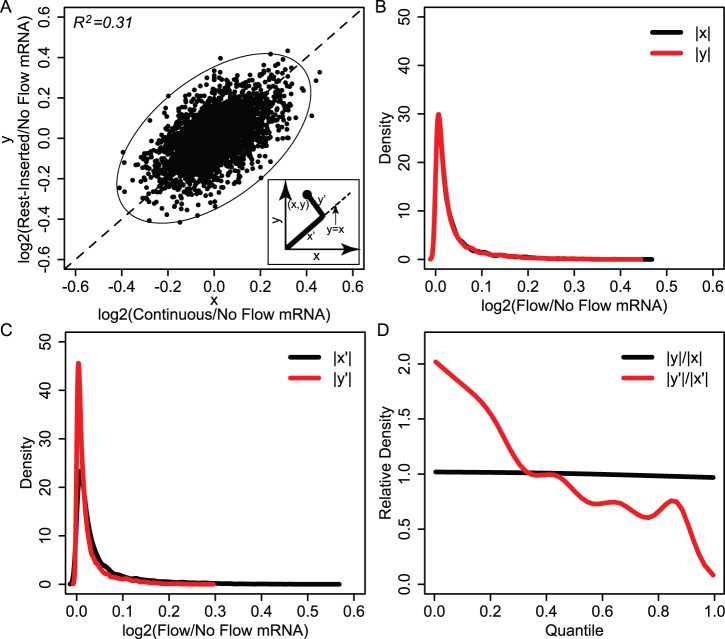
Genome-wide gene expression profiling reveals transcriptomic variations arising from rest-inserted flow. (A) Microarray gene expression data plotted in (x,y) space. Genes were distributed within an approximately elliptical-shaped region with major axis aligned parallel to y = x. The quantities x’ and y’ (inset) provide measures of common and differential gene expression alterations under the two flow conditions, respectively. (B–C) Density distributions for |x| and |y| (black and red lines respectively in B) and |x’| and |y’| (black and red lines respectively in C). (D) Relative distributions for |y|/|x| (black) |y’|/|x’| (red). For |y|/|x|, the distribution was essentially uniform and equal to one. This is in contrast to the relative distribution for |y’|/|x’|, which exhibited an inverse relation between relative density and quantile.

Given that temporal processing relies on the amplification of subtle variations in signaling network dynamics, we speculated that temporal variations in fluid flow would give rise to small perturbations in the expression of a large number of genes rather than large perturbations in a few genes. To assess this, we first visualized gene expression in (x,y) space (Fig. 3A). We observed that the genes were distributed within an approximately elliptical-shaped region with major axis aligned parallel to y = x. Importantly, genes appeared to be distributed as a single group of points, with no discernable points outside of this group that would be indicative of large perturbations in the expression of a few genes. We fit the boundaries of the data to an ellipse by computing the semi-major and semi-minor axes of the ellipse as x’_max_–x’_min_ = 0.52 and y’_max_–y’_min_ = 0.29 respectively. The ellipse defined the boundaries of an accessible gene expression space outside of which variations under the two flow conditions was not possible for the conditions used in this study. Given that *x*’ measures common gene expression under the two flow conditions, while *y*’ measures differential expression, the ellipse aspect ratio of ∼1.8 indicated that the range in variations in gene expression arising from the insertion of rest was approximately half of the range in common alterations arising from exposure to the two flow types.

Based on the above results suggesting that the range of differential expression arising from the insertion of rest intervals was smaller than the range of common expression induced by flow exposure (i.e., that the range of y’ was smaller than x’), we next sought to determine the degree to which the shapes of the distributions of y’ and x’ were different. In particular, we speculated that not only would the magnitudes of y’ be smaller than that of x’, but also that the distribution of y’ would be shifted toward smaller changes in a larger number of genes. To assess this, we computed the probability density functions for |*x*|, |*y*|, |x’|, and |y’|. More specifically, we computed the kernel density estimates for each quantity, resulting in a continuous distribution of the number of genes associated with each value. In doing so, we used the absolute values so that decreases and increases in expression were represented equivalently.

When we first compared the distributions of |*x*| and |*y*|, we found that they were nearly identical (Fig. 3B), with no significant difference between |*x*| and |*y*| as indicated by a Kolmogorov-Smirnov test (p = 0.15). This suggested that at the level of the genome, the gene expression distributions in response to rest-inserted and continuous flow were relatively indistinguishable. In contrast, when we compared the density functions for |*x*’| and |*y*’|, they were noticeably distinct (Fig. 3C), with the Kolmogorov-Smirnov test revealing a highly significant difference in the two distributions (p = 2.2×10^−16^). Further analysis revealed three distinct differences between |*x*’| and |*y*’|. First, in regard to range, the max value of |*x*’| was approximately twice that of |*y*’| (|*x*’|: 0.55, |*y*’|: 0.29), consistent with the aspect ratio of ∼2 we observed for the bounding ellipse in (*x*,*y*) space. Second, we found that the average value of |*x*’| was also approximately twice that of |*y*’| (|*x*’|: 0.047, |*y*’|: 0.025). This suggested that on average, the gene expression changes associated with exposure to rest were roughly half those associated with exposure to flow. Finally, we found that compared to |*x*’|, |*y*’| exhibited a higher density of genes at the lower quantiles, and lower density of genes at the higher quantiles. This was more apparent when we computed the relative density distributions [37] for |y’|/|x’| (i.e., the kernel density estimate for |y’| divided by that for |x’|) and |y|/|x| (Fig. 3D). In this case, for |*y*|/|*x*|, we found that the relative distribution was nearly uniform and approximately equal to one. This was in contrast to the relative distribution for |*y*’|/|*x*’|, which exhibited an inverse relation between relative density and quantile. In particular, for the lowest quantiles (i.e., genes which exhibited small changes), the density of |*y*’| was approximately twice that of |*x*’|; for the highest quantiles (i.e., genes which exhibited large changes), the density of |*y*’| was approximately one tenth of the density of |*x*’|. Collectively, these data suggested that the gene expression variations arising from the insertion of rest intervals (i.e., y’) differed from the common alterations under the two flow conditions (i.e., x’) in three distinct ways: 1) the magnitudes tended to be much smaller, 2) the range of variation was more narrow, and 3) the number of genes exhibiting variations at the upper end of this range was much more limited.

### Pathways Analysis of Transcriptomic Perturbations Implicates MAPK Signaling

**Figure 4:**
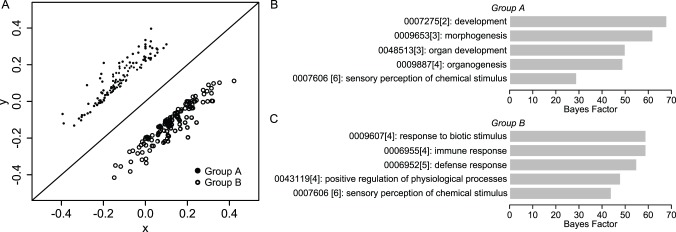
Establishment of Group A and B gene sets. (A) Visualization of Group A (black circles) and Group B (white circles), which consisted of genes with the top 1% of positive and negative values of y’ respectively. Physically, gene members of Group A and B were those that were the most differentially expressed and which were up-regulated and down-regulated in response to rest, respectively. (B–C) GO analysis results for Groups A and B. The most significant GO terms for Group A were associated with morphogenesis and developmental processes, while Group B terms were primarily associated with inflammatory and defense responses.

The above results suggested that the transcriptomic differences between continuous and rest-inserted flow were relatively subtle in magnitude. In this case, to identify biological mechanisms associated with the small gene expression perturbations arising from the insertion of rest intervals, we used over-representation analyses. Our use of this approach was motivated by the fact that over-representation enables the identification of biological associations independently of the specific levels of expression of each gene, making it ideal for investigating phenomena where subtle transcriptional variations in a large group of genes are expected [29]. For these analyses, we assessed two gene sets, Group A (120 genes) and Group B (119 genes), which consisted of genes with the top 1% of positive and negative values of y’ respectively (Fig. 4A). Our use of the top 1% as the cutoff was motivated by the fact that it enabled a group membership of ∼100 genes, a group size found to be efficacious for the pathways analysis tools utilized in this study [38]. Physically, Group A contained the most differentially expressed genes which were up-regulated in response to rest, whereas Group B contained the most differentially expressed genes which were down-regulated in response to rest.

To characterize the gene functions within Groups A and B, we first performed GO analysis. Analysis revealed 68 and 127 significant (i.e., Bayes factor >6 [29]) terms associated with Groups A and B, respectively. For simplicity, we analyzed the top five GO terms for each group, and found distinct functions associated with Group A and B gene signatures. In particular, Group A terms were primarily associated with morphogenesis and developmental processes (Fig. 4B), while Group B terms were primarily associated with inflammatory and defense responses (Fig. 4C). The fact that Group A and B gene signatures were associated with distinct functions suggested the potential for distinct causal signaling mechanisms to underlie their expression. To explore this question further, we analyzed Group A and B gene signatures using signaling pathway analysis as described below.

**Figure 5:**
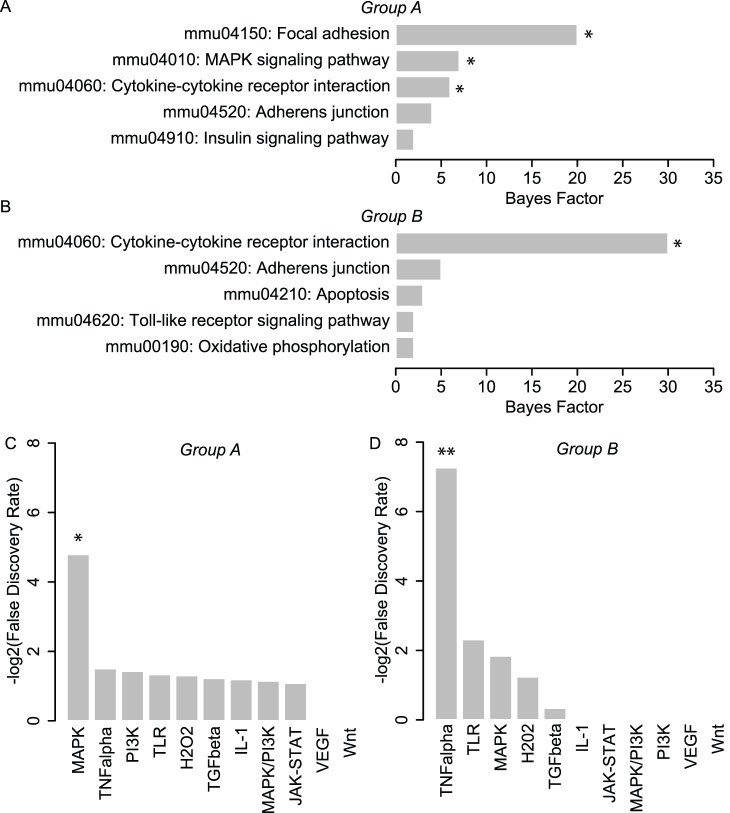
Pathways analysis of transcriptomic perturbations implicates MAPK signaling. (A–B): Results from KEGG analysis for Groups A and B. Group A was significantly associated with MAPK signaling (an intracellular signaling pathway) as well as two pathways associated with extracellular signal transduction: focal adhesion and cytokine-cytokine receptor interactions. Group B was significantly associated with cytokine-cytokine receptor interactions only. *Bayes Factor >6 (C–D): Results from SPEED analysis for Groups A and B. SPEED analysis revealed a significant overlap in Group A genes with the MAPK gene expression signature, and a significant overlap of Group B genes with the TNF-alpha pathway. *FDR <0.05.

We first explored pathway associations with Group A and B gene signatures using KEGG Pathway analysis. Our use of this approach was motivated by the comprehensiveness of the KEGG database, as well the large body of studies demonstrating its utility in mapping gene expression profiles to specific biological pathways. Using this approach, we found that Group A was significantly associated with MAPK signaling (an intracellular signaling pathway) as well as two pathways associated with extracellular signal transduction: focal adhesion and cytokine-cytokine receptor interactions (Fig. 5A). In addition, we found that Group B was significantly associated with cytokine-cytokine receptor interactions only (Fig. 5B). The fact that MAPK signaling was the only intracellular signaling pathway implicated in either group was of particular interest. In particular, these data suggested the potential involvement of the MAPK pathway in mediating rest-induced variations in the expression of Group A genes. However, it is important to note that the focus of KEGG analysis is to identify pathways perturbed as a consequence of altered expression of its components, not pathways responsible for driving their transcription [38]. Recognizing that signaling pathways often alter the expression of its own members through feedback regulation, association of a gene signature in KEGG analysis indirectly implicates it as a potential causal signaling pathway candidate [38], but does not infer a direct causal association. Thus, these results led us to pursue a more direct approach for assessing causal signaling pathways involved in driving Group A and B gene expression.

For causal pathway analysis, we analyzed Group A and B genes using SPEED [38], which enables gene signature associations to be revealed based on causal influences as opposed to pathway memberships alone [38]. The functionality of SPEED is rooted in the capacity for signaling pathways to regulate a conserved set of genes across diverse cell types and a broad range of experimental conditions, enabling comparison of user data with signature gene lists constructed from pathway perturbation experiments. Interestingly, SPEED analysis revealed a significant overlap in Group A genes with the MAPK gene expression signature (Fig. 5C), indicating that two functionally distinct approaches (causally-based SPEED analysis and membership-based KEGG analysis) associated MAPK signaling with Group A gene expression. SPEED analysis also revealed a significant overlap of Group B genes with the TNF-alpha pathway (Fig. 5D). To determine the sensitivity of MAPK and TNF-alpha association on the number of genes associated with each list, we iteratively increased the gene membership of Group A and B up to 200 genes and computed false discovery rate (FDR) for each 5 gene iteration. A significant association of MAPK for Group A was only observed for group sizes of 30 genes or greater, with the minimum FDR occurring when the group size was 50 genes. For Group B, a significant association of TNF-alpha was not observed until the group size was 115 genes, which also coincided with the minimum FDR within the range tested. The fact that a significant association for Group A was observed over a greater range of group sizes relative to Group B suggested that the association of the MAPK pathway was more robust to group membership size compared to TNF-alpha signaling. Taken together, the implication of MAPK signaling by two functionally distinct approaches for pathway analysis strongly suggested the involvement of this pathway in driving Group A gene expression. Based on these results, we focused on testing the predicted involvement of MAPK signaling.

### Rest-inserted Flow Enhances p-ERK1/2 Signaling by Enabling Recurring Activation

**Figure 6:**
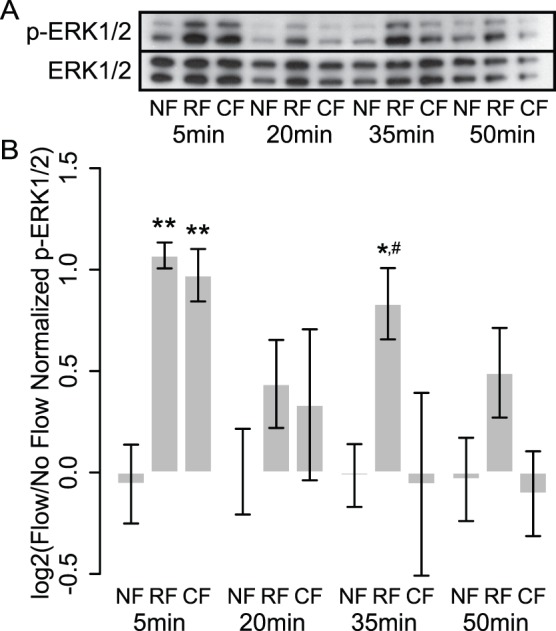
Rest intervals enhance flow-induced ERK1/2 activation by enabling recurring spikes in activity. (A) Western blot for p-ERK1/2 and ERK1/2 for cells subjected to no flow (NF), rest-inserted flow (RF), and continuous flow (CF) following cessation of each of the four flow bouts. (B) Quantification of Western blots from three separate experiments. Cells exposed to continuous flow exhibited a single spike in p-ERK1/2. In contrast, cells exposed to rest-inserted flow exhibited multiple spikes in p-ERK1/2 following each flow bout. We observed only modest increases in ERK1/2 activity after the second and fourth bouts but a significant increase after the third bout, suggesting that ERK1/2 may possess a refractory period of longer than 15 min but shorter than 45 min. *,**p<0.05, p<0.01 when compared to no flow at same time point, respectively. #p<0.05 when compared to continuous flow at same time point.

To confirm the predicted involvement of MAPK signaling in driving transcriptomic perturbations arising from rest, we assessed ERK1/2 phosphorylation dynamics in cells subjected to continuous and rest-inserted flow (profiles were identical to those in our gene expression studies). Our focus on ERK1/2 was motivated by its role as a ubiquitous and well-characterized MAP kinase associated with mechanotransduction [7]. We assessed p-ERK1/2 at four time points (5 min, 20 min, 35 min, and 50 min) corresponding to the cessation of each of the four rest-inserted flow bouts (Fig. 6). In cells exposed to continuous flow, we observed a single spike in p-ERK1/2, consisting of a rapid increase by 5 min, a decline by 20 min, and a return to baseline levels by 35 min. In contrast to the single spike in p-ERK1/2 during continuous flow, cells exposed to rest-inserted flow exhibited two significant spikes in p-ERK1/2, one during the first flow bout, and a second during the third flow bout (p = 0.007 and p = 0.019 for rest-inserted flow versus continuous flow and no flow, respectively). Interestingly, the activation following each flow bout was not uniform. In particular, though we observed modest increases in ERK1/2 activity after the second and fourth flow bouts, these increases were not significantly different from levels in cells exposed to continuous flow.

## Discussion

Modern gene expression profiling technologies have enabled an unprecedented opportunity to study cellular temporal processing from the viewpoint of the transcriptome. In this study, we used genome-wide gene expression profiling to identify and characterize acute transcriptomic differences in cells subjected to continuous versus rest-inserted mechanical stimulation. Our data establish, for the first time, the capacity for these gene expression variations to correctly identify acute signaling pathways underlying temporal processing in bone cells.

In assessing the potential for the recurring ERK1/2 activity identified in this study to occur in osteocytes residing within mechanically loaded bone, it is important to consider aspects of our model that may influence p-ERK1/2 dynamics. For example, we chose to use an osteoblastic cell line (MC3T3-E1) due to their extremely well-characterized signaling response under flow. However, a growing body of studies indicate that osteocytes and osteoblasts do not respond identically to flow exposure [39], suggesting that osteocytes may exhibit distinct p-ERK1/2 dynamics relative to osteoblasts. In addition, in our investigations we subjected cells to pulsatile flow using orbital shaking, which differs from the oscillatory waveform that is expected to occur *in vivo* [22]. While recent studies suggest that orbital flow and oscillatory flow give rise to similar bone cell responses for a range of outcomes [23], further studies are required to determine the degree to which these two flow profiles induce similar (or distinct) p-ERK1/2 dynamics in different bone cell lineages.

While the identified association between enhanced ERK1/2 activity and Group A gene expression was a direct result of our transcriptomic analysis, it should be recognized that this association does not imply causality. In particular, inferential pathway analysis tools like those used in our framework do not discriminate between associations that are causal or coincident. In our study, if signaling mechanisms upstream of ERK1/2 also regulate some or all of Group A genes via distinct pathways, then it is possible that ERK1/2 may have little or no direct effect on regulating Group A gene expression, despite the fact that it is functionally associated with the expression of these genes. While this does not detract from the utility of our approach for pathway discovery (generally, we are equally interested in identifying pathways that are both causal and coincident to gene expression changes), determining the physiological relevance of the MAPK pathway association identified here requires further study. For example, to determine the specific contribution of enhanced p-ERK1/2 in driving Group A gene expression, a potential strategy is to “normalize” the ERK1/2 activity of cells exposed to rest-inserted flow to that of cells exposed to continuous flow. This could be achieved by administering ERK1/2 inhibitors after the initial p-ERK1/2 spike (i.e., immediately after the first bout of rest-inserted flow) to inhibit any secondary spiking that occurs in subsequent flow bouts. Notably, the use of orbital shaking to generate flow is well-suited for such studies, as 6-well plates provide open access to cell media (and thus enable compound delivery in the absence of any further flow exposure).

It has been previously suggested that temporal processing during bone mechanotransduction may involve multiple pathways whose interactions are altered by the dynamics of the applied stimulus [12]. For example, both short- (0.5–14 s) and long- (0.5–8 hrs) duration rest intervals have been found to enhance loading-induced bone formation in rodents, with distinct time constants (i.e., relative gains in bone formation per unit time of rest) associated with their anabolic effects [12], [40]. This time dependence has been speculated to be due to the involvement of distinct mechanisms that are responsive to short- and long-duration rest periods [10]. In our studies, mechanically-stimulated ERK1/2 signaling had a refractory period of approximately 30 min (as evidenced by the lack of increase in p-ERK1/2 after the second and fourth flow bouts but a significant increase after the third bout), suggesting a potential role for recurring ERK1/2 activity in mediating enhanced anabolism arising from long-duration rest intervals. Interestingly, *in vitro*, short-duration (10 s) rest intervals have been found to elicit heightened intracellular Ca2+ mobilization in bone cells subjected to fluid flow [14]. In addition, previous studies suggest that flow-induced ERK1/2 activation occurs independently of intracellular Ca2+ mobilization in mechanically stimulated bone cells [41]. This suggests that enhanced Ca2+ signaling and recurring ERK1/2 activation may dually mediate temporal processing in a manner that is dependent on whether the rest intervals are short (10 s of seconds) or long (10 s of minutes) in duration. There is also the potential for other as of yet unidentified pathways to mediate temporal processing of intermediate rest durations (i.e., greater than 10 s but less than 30 min), an intriguing possibility that our framework is well-suited to explore.

While the focus of our study was on assessing the utility of transcriptomic perturbations arising from temporal variations in stimulation for pathway discovery, these data bring forth the question of whether such small transcriptomic perturbations may play a role in potentiating bone anabolism *in vivo*. In considering this question, it is notable that osteogenesis is mediated by a large number of genes interacting within a vast network. If this “osteogenic gene network” were to be considered as being analogous to a signaling network, its conductivity (e.g., the amount of bone formed per unit of stimulus) may be impacted to a much greater degree by small alterations in a large number of network components compared to a large change in any one component [42]. In this case, it is possible that small increases in the expression of a large number of osteogenic genes may have a much greater effect on bone formation relative to a large change in any single gene. However, regardless of their biological function, our studies suggest that identifying such transcriptomic perturbations within intact bone may have significant value from an analytical point of view. In particular, when integrated with systems-level analysis, such perturbations may be informative of acute mechanisms and causal signaling pathways enabling heightened bone anabolism in response to rest-inserted loading.

A unique aspect of our temporal processing analytical framework is its utility in revealing the emergence of different pathways in mediating responses to different rest period durations. In particular, by analyzing acute transcriptomic behavior, our framework indirectly probes a broad spectrum of signaling pathways rather than specific signaling pathway components. Importantly, this spectrum of pathways will only increase as new genome annotation databases and bioinformatics tools are developed. In addition, new technologies that enable high-throughput mechanotransduction studies [43], [44], [45] and transcriptomic analyses [46] are rapidly emerging. In this case, our studies may serve as the experimental foundation for future investigations that map the temporal trajectory of every single gene upon mechanical stimulation, and their variations in response to different rest intervals. Such explorations would have clear potential to yield fundamental insights into the mechanisms mediating temporal processing in bone and other tissues that exhibit this phenomenon [47], [48].

Several important limitations should be considered when interpreting the findings from this study. First, our use of the time-averaged profiles *x* and *y* likely reduced our sensitivity in identifying differentially regulated genes. In particular, genes that were differentially regulated at one time point but not the other were unlikely be assigned to Group A or B membership. However, it is important to note that our use of time-averaging likely enhanced the robustness of our predictions. In particular, Group membership was restricted to genes that exhibited sustained differences over both time points, which increased the selectivity of these groups. A second limitation is that our causal pathway analysis was performed using SPEED, which is limited to a relatively small number of pathways and uses data from experiments performed in human cells. However, we chose to utilize this approach due to the limited availability of tools for detecting causal signaling pathways and the fact that the majority of mouse and human genes are expected to have conserved biological function. Finally, while our studies demonstrated the ability for transcriptomic perturbations in cells subjected to rest-inserted fluid flow to correctly identify differential activation of the MAPK signaling pathway, it remains to be established whether our framework is able to correctly identify other signaling pathways in other experimental contexts. In this regard, the predicted association between TNF-alpha signaling and Group B gene expression provides a compelling starting point to explore this question further.

In summary, we characterized transcriptomic perturbations arising from the insertion of intermittent rest periods in bone cells subjected to fluid flow, and assessed the utility of these perturbations to identify signaling pathways that are differentially activated by this temporal variation. Our studies directly establish the capacity for transcriptomic perturbations arising from rest to correctly identify acute signaling pathways underlying these variations.

## Supporting Information

Table S1 **Primer sequences for real time RT-PCR.**
(DOC)

## References

[1] Mauk MD, Buonomano DV (2004) The neural basis of temporal processing. Annual Review of Neuroscience 27: 307–340. 10.1146/annurev.neuro.27.070203.144247 15217335

[2] Post R (1980) Intermittent versus continuous stimulation: effect of time interval on the development of sensitization or tolerance. Life Sciences 26: 1275–1282. 6991841 10.1016/0024-3205(80)90085-5

[3] Samaha A–N, Reckless GE, Seeman P, Diwan M, Nobrega JN, et al (2008) Less is more: antipsychotic drug effects are greater with transient rather than continuous delivery. Biological Psychiatry 64: 145–152. 18295747 10.1016/j.biopsych.2008.01.010

[4] Goddard GV, McIntyre DC, Leech CK (1969) A permanent change in brain function resulting from daily electrical stimulation. Experimental Neurology 25: 295–330. 4981856 10.1016/0014-4886(69)90128-9

[5] Frolik CA, Black EC, Cain RL, Satterwhite JH, Brown-Augsburger PL, et al (2003) Anabolic and catabolic effects of human parathyroid hormone (1–34) are predicted by duration of hormone exposure. Bone 33: 372–379. 13678779 10.1016/s8756-3282(03)00202-3

[6] Srinivasan S, Weimer DA, Agans SC, Bain SD, Gross TS (2002) Low-magnitude mechanical loading becomes osteogenic when rest is inserted between each load cycle. Journal of Bone and Mineral Research 17: 1613–1620. 12211431 10.1359/jbmr.2002.17.9.1613 PMC1435731

[7] Jacobs CR, Huang H, Kwon RY (2012) Introduction to Cell Mechanics and Mechanobiology. New York: Garland Science.

[8] Kwon RY, Meays DR, Tang WJ, Frangos JA (2010) Microfluidic enhancement of intramedullary pressure increases interstitial fluid flow and inhibits bone loss in hindlimb suspended mice. Journal of Bone and Mineral Research 25: 1798–1807. 20200992 10.1002/jbmr.74 PMC3153350

[9] Kwon RY, Meays DR, Meilan AS, Jones J, Miramontes R, et al (2012) Skeletal adaptation to intramedullary pressure-induced interstitial fluid flow is enhanced in mice subjected to targeted osteocyte ablation. PloS One 7: e33336. 22413015 10.1371/journal.pone.0033336 PMC3296683

[10] Sugiyama T, Saxon LK, Zaman G, Moustafa A, Sunters A, et al (2008) Mechanical loading enhances the anabolic effects of intermittent parathyroid hormone (1–34) on trabecular and cortical bone in mice. Bone 43: 238–248. 18539556 10.1016/j.bone.2008.04.012

[11] Umemura Y, Sogo N, Honda A (2002) Effects of intervals between jumps or bouts on osteogenic response to loading. Journal of Applied Physiology 93: 1345–1348. 12235034 10.1152/japplphysiol.00358.2002

[12] Robling AG, Burr DB, Turner CH (2001) Recovery periods restore mechanosensitivity to dynamically loaded bone. Journal of Experimental Biology 204: 3389–3399. 11606612 10.1242/jeb.204.19.3389

[13] LaMothe JM, Zernicke RF (2004) Rest insertion combined with high-frequency loading enhances osteogenesis. Journal of Applied Physiology 96: 1788–1793. 14707150 10.1152/japplphysiol.01145.2003

[14] Srinivasan S, Agans SC, King KA, Moy NY, Poliachik SL, et al (2003) Enabling bone formation in the aged skeleton via rest-inserted mechanical loading. Bone 33: 945–955. 10.1016/j.bone.2003.07.009 14678854

[15] Shinn JB (2003) Temporal processing: the basics. The Hearing Journal 56: 52.

[16] Hung CT, Pollack SR, Reilly TM, Brighton CT (1995) Real-time calcium response of cultured bone-cells to fluid-flow. Clinical Orthopaedics and Related Research: 256–269. 7641488

[17] Batra NN, Li YJ, Yellowley CE, You L, Malone AM, et al (2005) Effects of short-term recovery periods on fluid-induced signaling in osteoblastic cells. Journal of Biomechanics 38: 1909–1917. 16023480 10.1016/j.jbiomech.2004.08.009

[18] Donahue TLH, Haut TR, Yellowley CE, Donahue HJ, Jacobs CR (2003) Mechanosensitivity of bone cells to oscillating fluid flow induced shear stress may be modulated by chemotransport. Journal of Biomechanics 36: 1363–1371. 12893045 10.1016/s0021-9290(03)00118-0

[19] Donahue SW, Jacobs CR, Donahue HJ (2001) Flow-induced calcium oscillations in rat osteoblasts are age, loading frequency, and shear stress dependent. American Journal of Physiology - Cell Physiology 281: C1635–C1641. 11600427 10.1152/ajpcell.2001.281.5.C1635

[20] Kwon RY, Jacobs CR (2007) Time-dependent deformations in bone cells exposed to fluid flow in vitro: investigating the role of cellular deformation in fluid flow-induced signaling. Journal of Biomechanics 40: 3162–3168. 17559856 10.1016/j.jbiomech.2007.04.003 PMC2134832

[21] Thomas JMD, Chakraborty A, Sharp MK, Berson RE (2011) Spatial and temporal resolution of shear in an orbiting petri dish. Biotechnology Progress 27: 460–465. 21302366 10.1002/btpr.507

[22] Jacobs CR, Yellowley CE, Davis BR, Zhou Z, Cimbala JM, et al (1998) Differential effect of steady versus oscillating flow on bone cells. Journal of Biomechanics 31: 969–976. 9880053 10.1016/s0021-9290(98)00114-6 PMC3057628

[23] Young SRL, Hum JM, Rodenberg E, Turner CH, Pavalko FM (2011) Non-overlapping functions for Pyk2 and FAK in osteoblasts during fluid shear stress-induced mechanotransduction. Plos One 6: e16026. 21283581 10.1371/journal.pone.0016026 PMC3026802

[24] Ley K, Lundgren E, Berger E, Arfors KE (1989) Shear-dependent inhibition of granulocyte adhesion to cultured endothelium by dextran sulfate. Blood 73: 1324–1330. 2467707

[25] Berson RE, Purcell MR, Sharp MK (2008) Computationally determined shear on cells grown in orbiting culture dishes. Advances in Experimental Medicine and Biology 614: 189–198. 18290329 10.1007/978-0-387-74911-2_22

[26] Dardik A, Chen L, Frattini J, Asada H, Aziz F, et al (2005) Differential effects of orbital and laminar shear stress on endothelial cells. Journal of Vascular Surgery 41: 869–880. 15886673 10.1016/j.jvs.2005.01.020

[27] Livak KJ, Schmittgen TD (2001) Analysis of relative gene expression data using real-time quantitative PCR and the 2−ΔΔCT method. Methods 25: 402–408. 11846609 10.1006/meth.2001.1262

[28] Du P, Kibbe WA, Lin SM (2008) lumi: a pipeline for processing Illumina microarray. Bioinformatics (Oxford) 24: 1547–1548. 10.1093/bioinformatics/btn224 18467348

[29] Chang JT, Nevins JR (2006) GATHER: a systems approach to interpreting genomic signatures. Bioinformatics (Oxford) 22: 2926–2933. 10.1093/bioinformatics/btl483 17000751

[30] Zeng Y, Cowin SC, Weinbaum S (1994) A fiber-matrix model for fluid-flow and streaming potentials in the canaliculi of an osteon. Annals of Biomedical Engineering 22: 280–292. 7978549 10.1007/BF02368235

[31] You L, Cowin SC, Schaffler MB, Weinbaum S (2001) A model for strain amplification in the actin cytoskeleton of osteocytes due to fluid drag on pericellular matrix. Journal of Biomechanics 34: 1375–1386. 11672712 10.1016/s0021-9290(01)00107-5

[32] Anderson EJ, Kaliyamoorthy S, Alexander JID, Tate MLK (2005) Nano-microscale models of periosteocytic flow show differences in stresses imparted to cell body and processes. Annals of Biomedical Engineering 33: 52–62. 15709705 10.1007/s10439-005-8962-y

[33] Kwon RY, Frangos JA (2010) Quantification of lacunar-canalicular fluid flow through computational modeling of fluorescence recovery after photobleaching. Cellular and Molecular Bioengineering 3: 296–306. 21076644 10.1007/s12195-010-0129-8 PMC2976057

[34] Kwon RY, Temiyasathit S, Tummala P, Quah CC, Jacobs CR (2010) Primary cilium-dependent mechanosensing is mediated by adenylyl cyclase 6 and cyclic AMP in bone cells. FASEB Journal 24: 2859–2868. 20371630 10.1096/fj.09-148007 PMC2909282

[35] Chen NX, Ryder KD, Pavalko FM, Turner CH, Burr DB, et al (2000) Ca2+ regulates fluid shear-induced cytoskeletal reorganization and gene expression in osteoblasts. American Journal of Physiology - Cell Physiology 278: C989–C997. 10794673 10.1152/ajpcell.2000.278.5.C989

[36] Plunkett NA, Partap S, O’Brien FJ (2010) Osteoblast response to rest periods during bioreactor culture of collagen–glycosaminoglycan scaffolds. Tissue Engineering: Part A 16: 943–951. 19827912 10.1089/ten.TEA.2009.0345

[37] Handock MS, Morris M (1998) Relative distribution methods. Sociological Methodology 28: 53–97.

[38] Parikh JR, Klinger B, Xia Y, Marto JA, Bluthgen N (2010) Discovering causal signaling pathways through gene-expression patterns. Nucleic Acids Research 38: W109–W117. 20494976 10.1093/nar/gkq424 PMC2896193

[39] Kamel MA, Picconi JL, Lara-Castillo N, Johnson ML (2010) Activation of β-catenin signaling in MLO-Y4 osteocytic cells versus 2T3 osteoblastic cells by fluid flow shear stress and PGE2: Implications for the study of mechanosensation in bone. Bone 47: 872–881. 20713195 10.1016/j.bone.2010.08.007 PMC2952691

[40] Srinivasan S, Ausk BJ, Poliachik SL, Warner SE, Richardson TS, et al (2007) Rest-inserted loading rapidly amplifies the response of bone to small increases in strain and load cycles. Journal of Applied Physiology 102: 1945–1952. 17255366 10.1152/japplphysiol.00507.2006

[41] Liu D, Genetos DC, Shao Y, Geist DJ, Li J, et al (2008) Activation of extracellular-signal regulated kinase (ERK1/2) by fluid shear is Ca2+- and ATP-dependent in MC3T3–E1 osteoblasts. Bone 42: 644–652. 18291742 10.1016/j.bone.2007.09.058 PMC2937351

[42] Subramanian A, Tamayo P, Mootha VK, Mukherjee S, Ebert BL, et al (2005) Gene set enrichment analysis: A knowledge-based approach for interpreting genome-wide expression profiles. Proceedings of the National Academy of Sciences of the United States of America 102: 15545–15550. 16199517 10.1073/pnas.0506580102 PMC1239896

[43] Kim D-H, Wong PK, Park J, Levchenko A, Sun Y (2009) Microengineered platforms for cell mechanobiology. Annual Review of Biomedical Engineering 11: 203–233. 10.1146/annurev-bioeng-061008-124915 19400708

[44] Worton LE, Srinivasan S, Kwon RY (2013) Fluid dynamic gauging-based assays for high-throughput investigation of cellular mechanotransduction. 2013 Proceedings of the ASME Summer Bioengineering Conference.

[45] Riehl BD, Lim JY (2012) Macro and microfluidic flows for skeletal regenerative medicine. Cells 2012: 4. 10.3390/cells1041225 PMC3901127 24710552

[46] Wang Z, Gerstein M, Snyder M (2009) RNA-Seq: a revolutionary tool for transcriptomics. Nature Reviews Genetics 10: 57–63. 10.1038/nrg2484 PMC2949280 19015660

[47] Sen B, Xie Z, Case N, Styner M, Rubin CT, et al (2011) Mechanical signal influence on mesenchymal stem cell fate is enhanced by incorporation of refractory periods into the loading regimen. Journal of Biomechanics 44: 593–599. 21130997 10.1016/j.jbiomech.2010.11.022 PMC3042527

[48] Hanson AD, Marvel SW, Bernacki SH, Banes AJ, van Aalst J, et al (2009) Osteogenic effects of rest-inserted and continuous cyclic tensile strain on hASC lines with disparate osteodifferentiation capabilities. Annals of Biomedical Engineering 2009: 5. 10.1007/s10439-009-9648-7 19229619

