# Supplementary material for: Systems-Based Identification of Temporal Processing Pathways during Bone Cell Mechanotransduction
Source: PLoS One. 2013 Sep 11;8(9):e74205. doi: 10.1371/journal.pone.0074205 (PMC3770665; doi:10.1371/journal.pone.0074205)
Supplement: Table S1 — Primer sequences for real time RT-PCR. (DOC) [file pone.0074205.s001.doc]

| **Target** | **F (5’→3’)** | **R (5’→3’)** |
| --- | --- | --- |
| **Cyclophilin** | GCCGATGACGAGCCCTTGGGCC | ACCAGTGCCATTATGGCGTGTG |
| **β-actin** | TCACCCACACTGTGCCCATCTACGA | CAGCGGAACCGCTCATTGCCAATGG |
| **CFOS** | CGCAGAGCATCGGCAGAAGG | TCTTGCAGGCAGGTCGGTGG |
| **COX-2** | GCTGTACAAGCAGTGGCAAA | CCCCAAAGATAGCATCTGGA |
| **ICER** | TATGCAAAAGCCCAACATGG | GCGAGTTGCTTCTTCTGCTGCTAG |
| **Col1a1** | AATGGCACGGCTGTGTGCGA | AACGGGTCCCCTTGGGCCTT |
| **HO1** | CAAGCCGAGAATGCTGAGTTCATG | GCAAGGGATGATTTCCTGCCAG |
| **OPN** | TGCACCCAGATCCTATAGCC | CTCCATCGTCATCATCATCG |
| **IL6** | AGTTGCCTTCTTGGGACTGA | CAGAATTGCCATTGCACAAC |
| **DKK2** | CCGCTGCAATAATGGAATCT | GTAGGCATGGGTCTCCTTCA |
| **RANKL** | CCAAGATCTCTAACATGACG | CACCATCAGCTGAAGATAGT |
| **FGF23** | AGTCGGTTCAGCCCACGTAGAGGA | TGAATAGCGGTGCCTGGCTGGA |
| **NOS2** | CCTTCCTTGCATGTGCCCGCT | CCCTGACCATCTCGGGTGCG |
| **OSX** | CCCTTCTCAAGCACCAATGG | AGGGTGGGTAGTCATTTGCATAG |
| **P2XR7** | CTGTGGTCTAGCCTGGGAAG | TCTTTCCGCTGGTACAGCTT |
| **VEGF** | GAGAGAGGCCGAAGTCCTTT | TTGGAACCGGCATCTTTATC |
| **SMAD5** | CCGATTCTTTCCACCAACC | GCTGGGGAGTTGGGATATGT |
| **TGFβ1** | TGTCGCTCTCGGGTCACGGA | GTTGTCTCCCCAGCCGGCAC |
| **DSCR1** | CCGTGTGGAATTGTCCTTCT | ACTTCTTTTGCAGGGAAGCA |
| **TNFα** | AGCCCCCAGTCTGTATCCTT | CTCCCTTTGCAGAACTCAGG |
| **OPG** | CTGCCTGGGAAGAAGATCAG | TTGTGAAGCTGTGCAGGAAC |
| **RUNX2** | AAGTGCGGTGCAAACTTTCT | TCTCGGTGGCTGGTAGTGA |
